# The Takeout Restaurant Foods Blood Glucose Monitoring Method: A New Educational Approach to Nutritional Guidance for Dining Out

**DOI:** 10.7759/cureus.77252

**Published:** 2025-01-10

**Authors:** Kyoko Tajima, Miyu Chinen, Atsushi Iha, Yuzuru Ohshiro

**Affiliations:** 1 Department of Nutrition, Omoromachi Medical Center, Naha City, JPN; 2 Department of Physical Therapy, Omoromachi Medical Center, Naha City, JPN; 3 Department of Internal Medicine, Omoromachi Medical Center, Naha City, JPN

**Keywords:** blood glucose monitoring, nutritional guidance, obesity, patient education, post-prandial blood glucose

## Abstract

This report introduces a novel approach to providing nutritional guidance for people dining out, utilizing takeout meals as a practical tool. The method comprises several essential steps: 1) Preparing takeout versions of restaurant dishes and bringing them to the hospital, 2) performing comprehensive nutritional evaluations of these meals and adjusting them as necessary to meet specific dietary needs, and 3) assessing the impact of these modified meals on post-meal blood glucose levels. This assessment is achieved through continuous blood glucose monitoring at crucial time points: before the meal, 60 minutes after beginning the meal, and 120 minutes after eating. Traditional methods of nutritional guidance for dining out, often based on showing textual photos or gathering information on the dining out habits of patients, face challenges in consistency and reliability due to variations in meal composition and nutritional values. This approach focuses on direct interaction with actual meals and real-time blood glucose monitoring, offering a more specific and effective way to provide nutritional advice. Not only is it suitable for hospital settings, but its simplicity and practicality also make it adaptable to a variety of medical facilities. This approach aids in blood glucose control for patients and also provides valuable feedback to educators, thus contributing to the enhancement of their skills.

## Introduction

In developed countries, there has been an increase in dining out. The United States population, for instance, obtains approximately 20% of their total calories from dining out [1]. Dining out often leads to excessive calorie intake, contributing to obesity, a significant health concern in many countries [1,2]. Moreover, individuals with diabetes are susceptible to elevated blood glucose levels due to excessive carbohydrate consumption. Increased postprandial blood glucose levels have been linked to higher cardiovascular risk, regardless of fasting plasma glucose or hemoglobin A1c (HbA1c). Some studies suggest that improved postprandial glucose control can mitigate cardiovascular risk [3-5]. Nutritional instructions for eating out commonly rely on model meal descriptions and photographs [6-9]. However, variations in cooking oil quantities and other factors among different restaurants can result in substantial differences in calorie and nutrient content for the same menu items. Providing uniform guidance based on model meals is challenging. Efforts involving food item photos and guidance have been made to address individual dining choices. Nevertheless, difficulties persist in estimating portion sizes and seasoning oil amounts when analyzing food photos. Furthermore, the issue of not being able to understand what effects such dining-out guidance might have remains a challenge. As a result, traditional dining-out guidance has often struggled to demonstrate measurable effects. To address these challenges, our diabetes care team at the hospital implemented an innovative strategy where patients brought their takeout meals to the clinic for personalized guidance. This method enabled more accurate nutritional assessments through direct interaction with real food items. Moreover, we assessed the impact of nutritional adjustments by continuously monitoring blood glucose levels before and after guidance. This approach not only allows for precise nutritional evaluations using actual food but also permits us to gauge the effectiveness of dietary modifications by tracking blood glucose responses. This improvement in blood glucose levels enables patients to understand the effects of nutritional interventions. This straightforward approach can be readily adopted not only in hospitals but also in various clinics. We believe that this method holds significant promise as a valuable strategy in diabetes education.

## Case presentation

Method

Patients are requested to bring takeout meals from their regular dining establishments to the hospital. A nutritionist evaluates the nutritional content, including calorie count, of the provided food items. Following this evaluation, patients consume a meal at the hospital, and their blood glucose levels are monitored to assess the impact on post-meal blood glucose levels (pre-meal, 60 minutes after meal initiation, and 120 minutes after). Monitoring of blood glucose levels employs a simple blood glucose meter (Medisafe kit, Terumo, Tokyo, Japan).

One week later, the identical meal is retrieved for takeout, and nutritional adjustments are implemented in accordance with the physician's recommendations. These modifications may encompass the reduction of specific food portions. In the wake of these dietary adjustments, blood glucose monitoring persists, utilizing the same blood glucose meter (Medisafe), at the same intervals spanning meals.

Cases and results

Case 1

**Figure 1:**
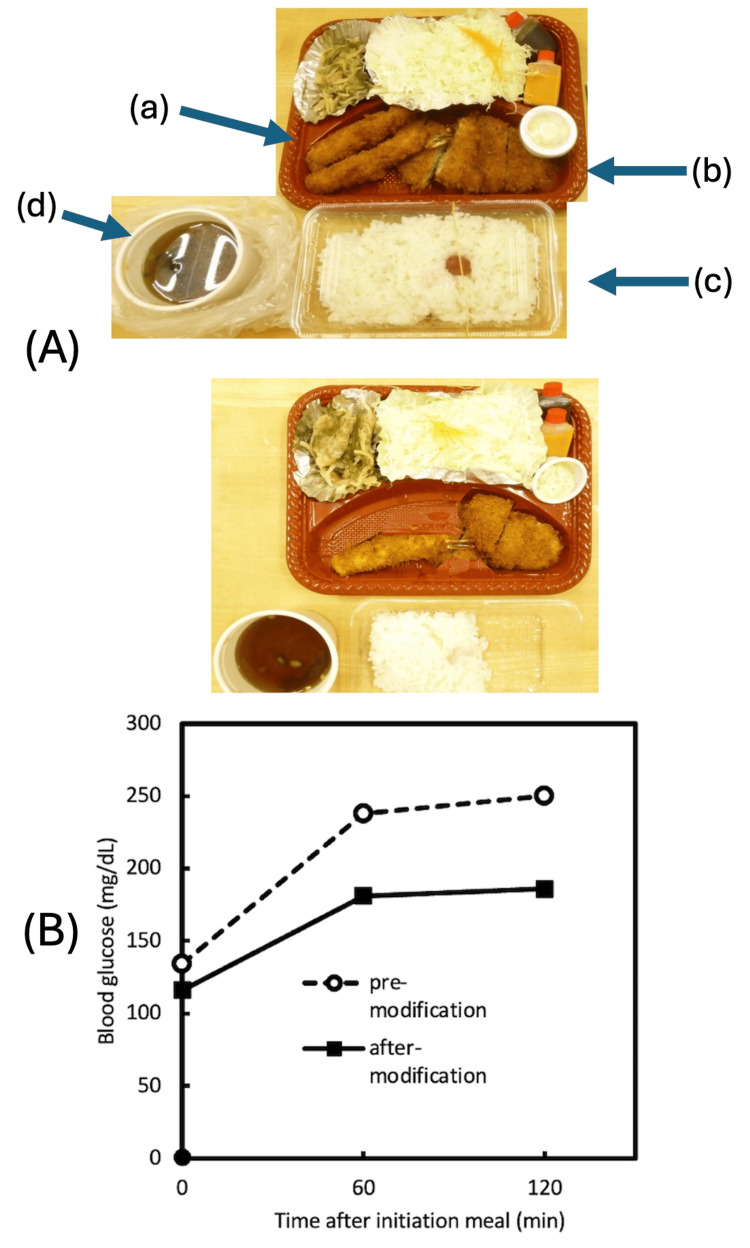
Nutritional Intervention and Improvement in Postprandial Blood Glucose in Case 1 (A) A photograph of the nutritional intervention. The upper part shows the meal before the intervention, and the lower part shows the meal after the intervention. The intervention consisted of the following changes: (a) reducing the number of fried shrimp from two to one, (b) reducing the pork cutlet to three-quarters, (c) reducing the portion of rice to two-thirds, and (d) reducing the portion of miso soup to two-thirds. (B) A graph showing blood glucose levels monitored from before a meal to after a meal, both before and after the nutritional intervention.

A male patient in his 70s with type 2 diabetes had a meal at the hospital, and his blood glucose levels were monitored. The calorie content of the restaurant meal brought for takeout was 1300 kcal. A week later, the same meal was brought for takeout, and nutritional adjustments were made. After the adjustments, the calorie content was reduced to 620 kcal (as shown in Figure 1A). As a result of these changes, there was an improvement in blood glucose levels (as shown in Figure 1B).　

Case 2

**Figure 2:**
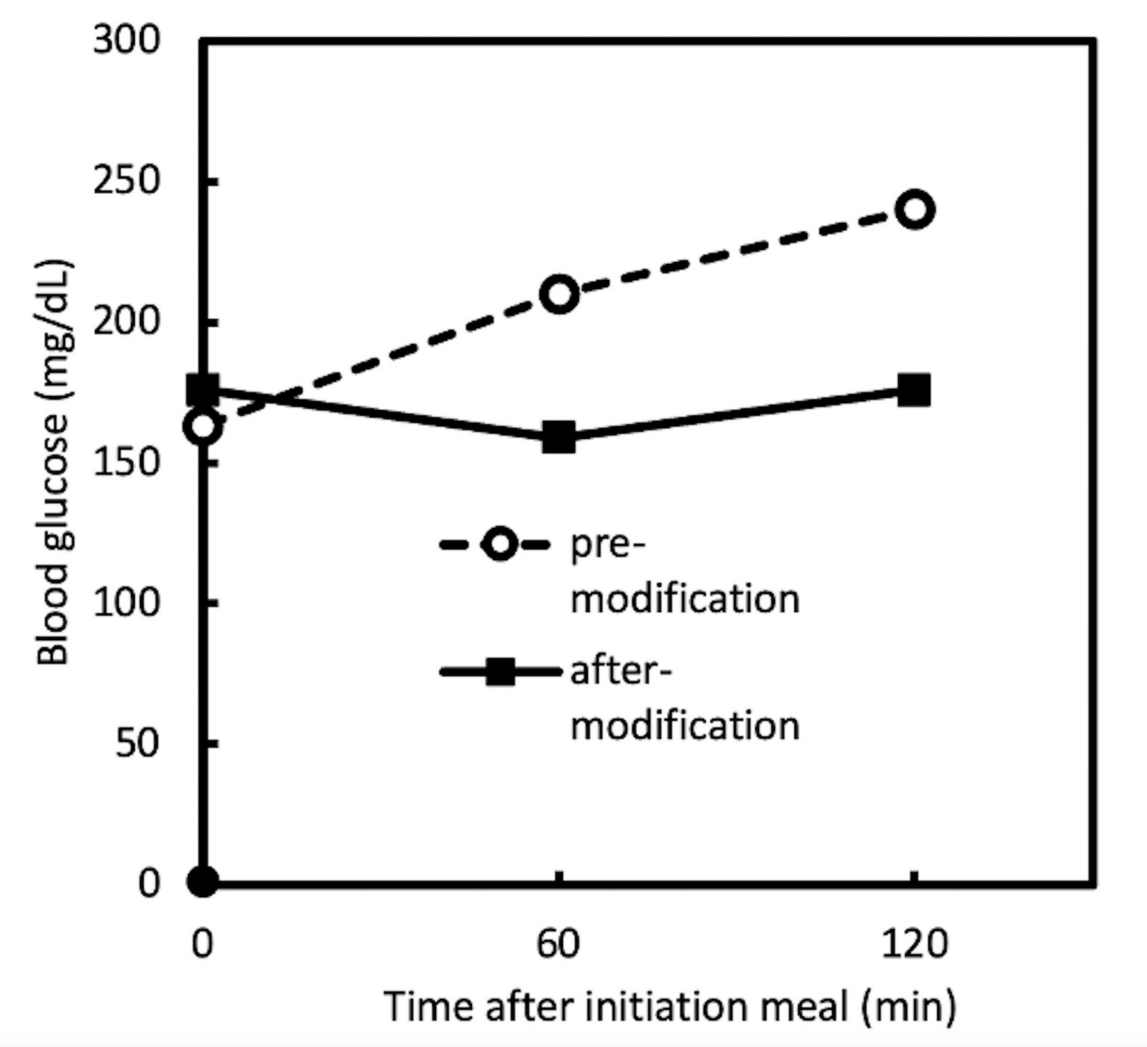
Improvement in Postprandial Blood Glucose in Case 2 A graph showing blood glucose monitoring from before a meal to after a meal, both before and after the nutritional intervention.

Case 2 was a male patient in his 50s with type 2 diabetes. The calorie content of the restaurant meal brought for takeout was 2000 kcal. He had a meal at the hospital, and his blood glucose levels were monitored. A week later, the same meal was brought for takeout, and nutritional adjustments were made. After nutritional adjustments, it was reduced to 620 kcal. Blood glucose levels improved after the nutritional changes (Figure 2).

## Discussion

The innovative approach presented in this report addresses the limitations of traditional nutritional guidance for dining out. Based on our search of several databases, including PubMed, Embase, Scopus, and Google Scholar, we could not find any previous reports documenting this approach. While conventional methods have focused on generalized advice such as portion control, menu selection, substitutions, and avoiding high-calorie items, these approaches can be imprecise and challenging to apply to individual patient scenarios [10]. Even efforts to incorporate food photographs have fallen short of accurately assessing nutritional content, especially when it comes to factors like portion sizes and cooking methods [8,9].

In contrast, our strategy leverages the tangible aspect of real takeout meals, allowing for direct evaluation of the actual food consumed. This not only ensures more accurate nutritional assessments but also facilitates personalized guidance tailored to the specific meal brought in by each patient. The subsequent monitoring of blood glucose levels before and after implementing nutritional adjustments provides a concrete measure of the impact on postprandial glucose responses. This approach bridges the gap between theoretical nutritional recommendations and their real-world effects on patients' metabolic outcomes.

The presented cases demonstrate the efficacy of this approach in achieving improved blood glucose control through targeted nutritional adjustments. The significant reduction in calorie content while maintaining meal satisfaction showcases the potential for enhancing dietary choices without sacrificing enjoyment. Moreover, the simplicity of this method enables its widespread adoption, not only within hospitals but also in various medical settings. Its practicality extends its utility beyond diabetes education to broader applications, where postprandial glucose management is essential.

Despite an exhaustive search of major databases, no prior reports detailing this specific approach were found. This underscores the novelty of our method, which contributes a valuable addition to the field of diabetes care and nutritional counseling. By emphasizing real-food interactions, our approach aligns with the growing trend toward personalized medicine, catering to the unique needs and preferences of each patient.

While this approach shows promise, there are some limitations to consider. The study's sample size is small, and a more extensive patient population is needed to establish its generalizability. Additionally, factors such as individual variability in metabolism and response to dietary changes might influence outcomes. Long-term studies could provide insights into the sustained effects of the proposed method and its impact on overall glycemic control and cardiovascular risk factors.

## Conclusions

The approach detailed in this report offers a novel, practical, and personalized method for nutritional guidance when dining out. By utilizing real takeout meals and monitoring blood glucose responses, it bridges the gap between theoretical recommendations and tangible outcomes. As an innovative tool for diabetes education, this method holds potential not only for improving blood glucose control but also for fostering a deeper understanding of the intricate relationship between diet and metabolic health. Further research and adoption across medical facilities can validate its effectiveness and broaden its impact on patient care.
